# The Psychological Effect of Excessive Gingival Display on Egyptian Females

**DOI:** 10.1155/tswj/9996803

**Published:** 2025-10-23

**Authors:** Lubna Ahmad Amro, Mahetab Mohammed Abdalwahab, Nada Zazou, Ahmed Elsayed Hamed Amr

**Affiliations:** ^1^Department of Oral Medicine, Periodontology and Oral Diagnosis, Faculty of Dentistry, October University for Modern Sciences and Arts, Giza, Egypt; ^2^Department of Oral Medicine, Periodontology and Oral Diagnosis, Faculty of Dentistry, Ain Shams University, Cairo, Egypt

**Keywords:** excessive gingival display, gummy smile, oral health impact factor, oral health–related quality of life

## Abstract

**Purpose:**

The purpose of this study is to evaluate the effect of excessive gingival display on oral health–related quality of life of Egyptian females.

**Methods:**

This cross-sectional study was conducted on 160 individuals with excessive gingival display and 160 controls matched for gender and age, attending the outpatient clinic. The outcome was oral health–related quality of life evaluated using the Oral Health Impact Profile-14 (OHIP-14) questionnaire.

**Results:**

Participants with excessive gingival display had a higher total OHIP-14 score (6.37 ± 3.34) in comparison with the controls (3.68 ± 2.54, *p* = <0.001). Impacts were also significant in the domains: functional limitation, physical disability, psychological discomfort, and psychological disability. Mean gingival display on maximum smile in the excessive gingival display group was 3.84 ± 1.13 mm.

**Conclusion:**

Excessive gingival display negatively affects the overall oral health–related quality of life, especially the psychological domain in this particular population. The results of this present study justify the correction of excessive gingival display to improve individuals' oral health–related quality of life.

## 1. Introduction

An esthetic smile is defined as the perfect balance between three parameters: the white (teeth), the pink (gum), and the lips [1]. Gingival display is gum exposure between the inferior border of the upper lip and the gingival margin of the maxillary anterior central incisors when smiling. Gingival exposure of more than 2 mm when a person smiles is considered an excessive gingival display or gummy smile [2, 3]. Gingival excess is classified by the American Academy of Periodontology (2017) as a mucogingival deformity and condition around the teeth and under “other conditions affecting the periodontium” [4]. Excessive gingival display has been a cause of embarrassment for many patients, often associated with refusal to smile, thus affecting their psychosocial behavior and leading to lack of self-esteem and challenges in social relations [5]. Some studies reported that 0–2 mm gingival display upon smiling was considered attractive, while between 2 and 3 mm display was considered less attractive [6–8]; the more the display, the less the attractiveness score [9]. Another study found that the amount of gingival display was inversely related to how friendly, trustworthy, intelligent, and self-confident a person is perceived by laymen [10]. Excessive gingival display is a clinical condition that has gained tremendous focus recently and is a chief complaint of many patients, perhaps because of the media and increasing emphasis on beauty standards [11]. Esthetic demands of the Egyptian patient have increased over the past two decades because modern technology has become available and exposure of the citizens to universal esthetic standards has increased, raising their awareness about oral health [12].

The World Health Organization defined oral health as “oral health that enables an individual to speak, eat and socialize without active disease, discomfort or embarrassment.” The Oral Health Impact Profile-49 (OHIP-49) questionnaire is considered an instrument that measures people's perception of the social impact of oral disorders on their well-being based on the theoretical model of oral health, which included seven dimensions: functional limitation, physical pain, psychological discomfort, physical disability, psychological disability, social disability, and handicap [13]. Responses were made on a Likert-type scale and coded 4 = *very often*, 3 = *fairly often*, 2 = *occasionally*, l = *hardly ever*, and 0 = *never*. However, the earliest version consisted of 49 questions and its implementation in the day-to-day clinical practice or in large-scale studies was quite difficult, and authors needed a more concise version [14]. The Oral Health Impact Profile-14 (OHIP-14) questionnaire, which consisted of only 14 questions to evaluate the oral health–related quality of life, was proposed to make implementation of the questionnaire more practical [15]. Since its publication in English in Australia, the OHIP-14 has been translated into different languages, including Chinese, Spanish, Danish, Portuguese, and Finnish [16–20].

Prevalence of excessive gingival display was found to be 10% in the population between the ages of 20 and 30 years, with statistically significant gender difference in prevalence and extent of display, and higher in females, respectively [2, 21, 22]. Prevalence of gummy smile in the Egyptian population was 9% in 2005 [23], and about a decade later, another study by the same authors found the prevalence of gummy smile to be 11.8% [12]. A more recent study found the prevalence of excessive gingival display to be 10.9% in the Turkish population [24]. Therefore, excessive gingival display is quite a common finding, affecting a great number of people worldwide. This study is aimed at examining the effect of excessive gingival display on the psychological aspect of oral health–related quality of life, in hopes that this would allow clinicians to better understand their patients' needs and provide patient-centered care, tailored to each patient's needs.

## 2. Subjects and Methods

### 2.1. Sample Size Calculation

According to Antoniazzi et al. [25] and based on the distribution of the Egyptian population from https://www.capmas.gov.eg/ (2021), a sample size of 160 patients for each group was sufficient to detect the effect size, estimate a 95% confidence interval, and achieve a significant level of 5% (*p* < 0.05). The sample size was calculated according to G∗Power software Version 3.1.9.4, where fS is the effect size, *α* = 0.05, *β* = 0.2, and Power = 1 − *β* = 0.95.

### 2.2. Study Design

**Figure 1:**
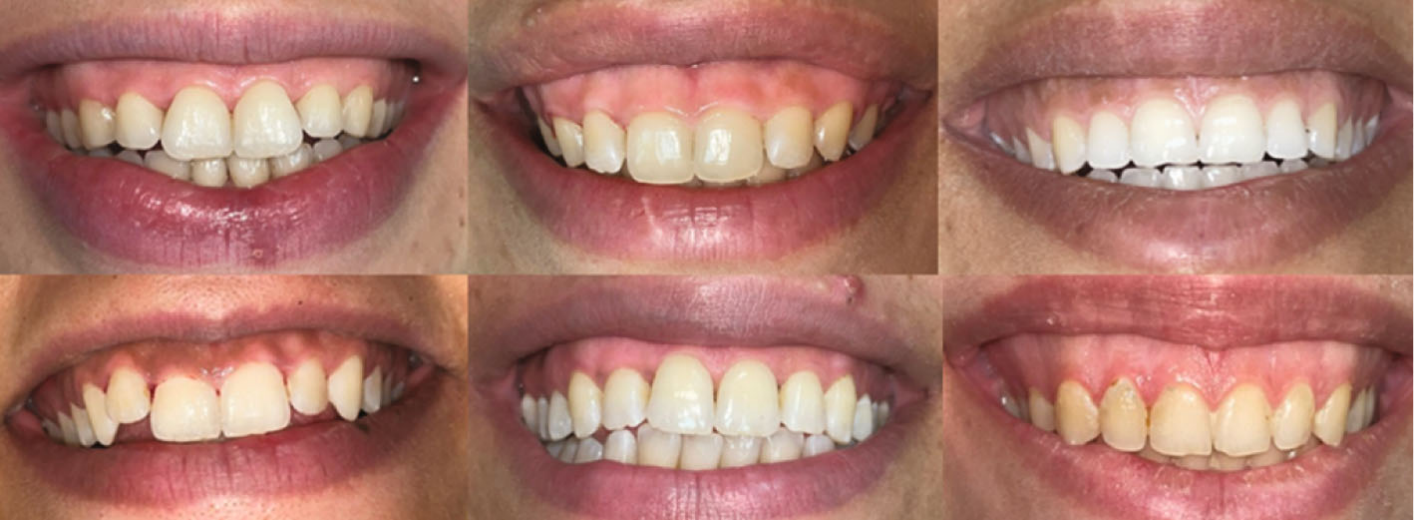
Excessive gingival display upon maximum smile of some candidates included in this study.

One hundred and sixty medically free female patients between age 18 and 40 years who had excessive gingival display and 160 controls matched in age and gender were recruited for this cross-sectional study from patients attending the outpatient clinic of the Oral Medicine, Periodontology and Oral Diagnosis Department, Faculty of Dentistry, Ain Shams University and patients attending the outpatient clinic of the Oral Medicine and Diagnosis department, Faculty of Dentistry, MSA University from September 2022 to December 2023. Subjects with periodontitis, badly decayed and mutilated anterior teeth, or any other intraoral finding that may affect their perception of oral health were excluded from this study. Excessive gingival display was diagnosed as more than 2 mm maxillary gingival display on maximum smiling, taken by calculating the average of the display associated with the right and left upper central incisors (Figure 1).

**Figure 2:**
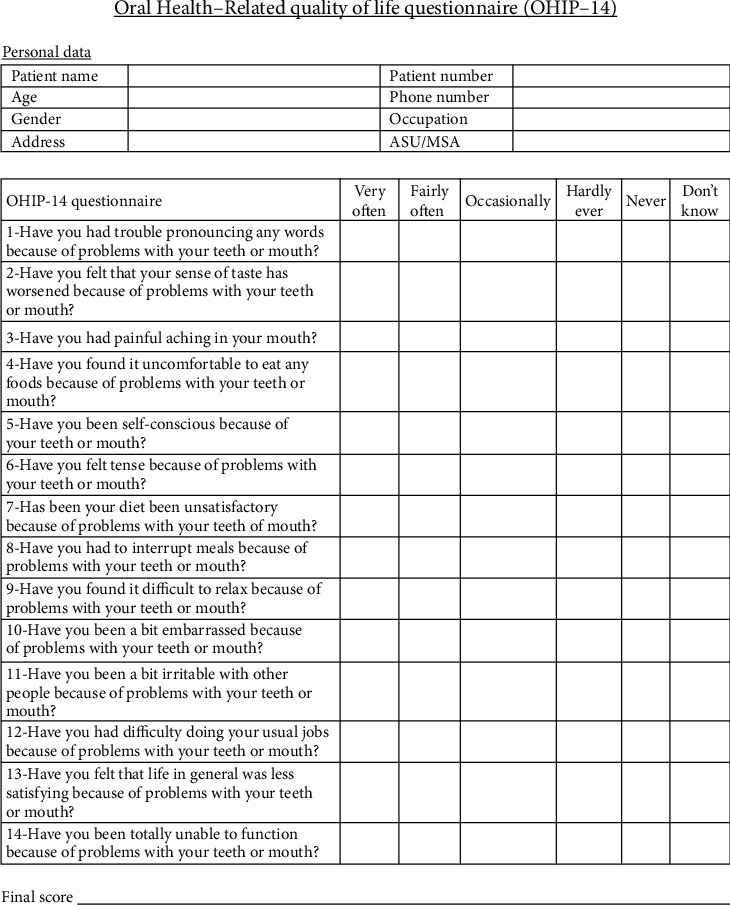
OHIP-14 questionnaire [15].

The measurements in this study were taken using a North Carolina Periodontal Probe-15 and a digital caliper. Dimensions of upper central, lateral, and canine were measured, and the recurring esthetic dental proportion was calculated. Subjects recruited in the excessive gingival display group were aware or made aware that they had excessive gingival display. Afterwards, patients were asked to take the OHIP-14 questionnaire (Figure 2) that had been translated into Arabic, as an instrument to evaluate their oral-related quality of life. This study did not contain illiterate patients. The same interviewer conducted all the interviews, patients filled out the data themselves, and the interviewer explained any questions they did not find clear enough. OHIP-14 questionnaire responses were made on a Likert-type scale and coded 4 = *very often*, 3 = *fairly often*, 2 = *occasionally*, l = *hardly ever*, and 0 = *never*, with the lowest total score 0 and the highest possible score 56. Participants in both groups were selected very carefully, ensuring that they were medically free females, aged 18–40 years, with overall good oral hygiene. Mild gingivitis was permitted, but cases with extensive periodontal disease were excluded. Participants had sound anterior teeth, especially from right canine to left canine; crowns and mutilated anterior teeth were also excluded—all in an effort to evaluate the sole effect of excessive gingival display on candidates' self-esteem/confidence without being affected by any other oral/dental esthetic problems (see S1).

This cross-sectional study was conducted as per the guidelines of strengthening the reporting of observational studies in epidemiology (STROBE) (Von Elm et al., 2007). The study was reviewed and approved by the Research Ethical Committee of the Faculty of Dentistry at Ain Shams University (approval number: FDASU-Rec IM122107; approval date: 22/12/21). A written informed consent form was read, understood, and signed by all the participants. All questionnaires were answered, and no fields were left empty. The sample was representative of the reference population.

### 2.3. Statistical Analysis

The mean and standard deviation values were calculated for quantitative data, while frequencies were calculated for qualitative data. Fisher's exact and chi-square tests were used to determine the relationship between frequencies. The significance level was set at *p* ≤ 0.05. Mann–Whitney *U* test was used because the data was not normally distributed. Spearman's test was used to determine correlation. Statistical analysis was performed with IBM SPSS Statistics Version 20 for Windows.

## 3. Results

A total of 320 individuals were included in this study, 160 with excessive gingival display and 160 without excessive gingival display. Thirty-two candidates who met the criteria refused to participate due to embarrassment at smiling or refusal to be photographed. Mean age of the excessive gingival display group was 27.62 ± 6.21, and mean age of the control group was 27.06 ± 6.06, *p* = 0.287 (Table 1). Mean gingival display on maximum smile in the excessive gingival display group was 3.84 ± 1.13 mm. The average display considered that unesthetic is between 2 and 3 mm [8]. OHIP-14 questionnaire responses were made on a Likert-type scale and coded 4 = *very often*, 3 = *fairly often*, 2 = *occasionally*, l = *hardly ever*, and 0 = *never*, with the lowest total score 0, and the highest total score in this study was 16 and 12 for the excessive gingival display and the control groups, respectively. Participants with excessive gingival display had a higher total OHIP-14 score (6.37 ± 3.34) in comparison with the controls (3.68 ± 2.54, *p* ≤ 0.001). Impacts were also significant in the domains: functional limitation, physical disability, psychological discomfort, and psychological disability (Table 2) (see S1 and S2).

**Figure 3:**
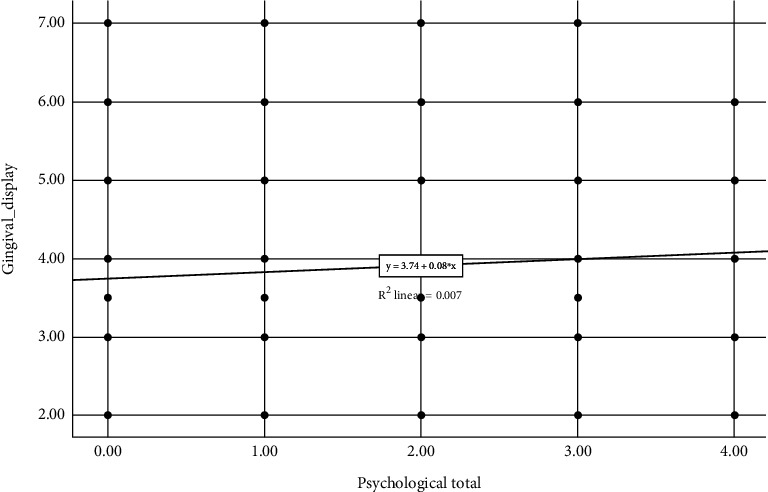
Scatter plot representing correlation between the extent of gingival display and the total psychological dimension (psychological discomfort + psychological disability) of OHIP-14 (see S2).

Spearman's correlation identified a minimal positive relationship between the extent of gingival display on maximum smile and the total psychological dimension of OHIP-14, which means that an increase in gingival display on maximum smile is accompanied by an increase in total psychological dimension with a correlation coefficient of 0.076 (Figure 3). Moreover, the correlation coefficients of psychological disability and psychological discomfort were 0.059 and 0.108, respectively (see S2).

## 4. Discussion

Smiles are a very important mode of nonverbal communication, conveying friendliness, approachability, and happiness and influencing people's perception of facial esthetics. The findings of this current study indicate that excessive gingival display upon smiling negatively influences an individual's oral health–related quality of life. Little data has been available on the individual's perception of oral health–related quality of life in relation to excessive gingival display.

Among the limitations of this study was that it was performed on a predominantly female sample; the authors chose this study design because several previous studies found a significant difference between genders that may affect results, since the prevalence of gummy smile was much higher in females than in males [2, 21, 22]. Moreover, many studies that had a sample consisting of both genders had more female candidates, which probably influenced the results [26–29]. In addition, females were more likely to smile than males [30]. Also, females are generally more aware of the appearance of their smile, more psychologically affected, and more committed when it comes to oral health [31]. The ages of the samples were 18–40, with a mean age of 27.62 ± 6.21 years in the excessive gingival display group and 27.06 ± 6.06 (*p* = 0.287) in the control group. Even though a gummy smile can be present at all ages, this particular age group was chosen because younger individuals and especially females are among the most psychologically affected groups by beauty standards in terms of self-esteem and confidence [32, 33] and because muscle flaccidity related to aging decreases the incidence of gummy smile in older individuals [34]. Another limitation is the use of convenience sampling, which may cause potential selection bias, but because of the cross-sectional study design, examination of association is done, not causality.

The gingival display on maximum smile mean value was 3.84 ± 1.13 mm in the excessive gingival display group. The average display considered unesthetic is between 2 and 3 mm [8]. A similar cross-sectional study conducted on 200 high school females aged 16 to 18 with excessive gingival display found a mean display of 4.68 ± 1.2 mm [35]; the difference in mean gingival display may be due to the wider age range, which included older individuals, and gingival display decreases with age [34]. The interviewer in this study noticed that many participants, when asked to smile, instinctively covered their mouths with their hands at first, but when asked to fill in the survey questions, they responded *never* to most of them, which was quite puzzling. Thirty-two candidates who met the criteria refused to participate due to embarrassment at smiling or refusing to be photographed.

The mean value for OHIP-14 total score was 6.37 ± 3.34 and 3.68 ± 2.54 (*p* ≤ 0.001) in the excessive gingival display and control groups, respectively. The meticulous selection of candidates explains the overall low means; other epidemiological studies that have used the OHIP have found that missing teeth, untreated decay, periodontal attachment loss, and barriers to dental care are associated with increasing scores, which were not included in the sample of this current study [14, 15, 36].

A study that was conducted in Southern Brazil using the OHIP-14 questionnaire aimed to compare the oral health–related quality of life between individuals with and without excessive gingival display and found that individuals with excessive gingival display did have a poorer oral health–related quality of life than individuals without excessive gingival display [25]. Total OHIP-14 scores were 2.10-fold higher among individuals with excessive gingival display; participants with excessive gingival display had a higher total OHIP-14 score (4.81 ± 4.76) compared to the controls (1.85 ± 3.77; *p* < 0.001), which is close to the findings of this study 6.37 ± 3.34 and 3.68 ± 2.54 (*p* < 0.001), respectively. Perhaps the higher value in this current study was because the sample comprised females only, who are more aware and affected by excessive gingival display than their male counterparts. In addition, this current study included patients undergoing orthodontic treatment, while Antoniazzi et al. excluded patients undergoing orthodontic treatment [25]. Antoniazzi et al. found that the domains significantly affected in the OHIP-14 by excessive gingival display were functional limitation (*p* < 0.001), psychological discomfort (*p* = 0.001), psychological disability (*p* = 0.032), and social handicap (*p* = 0.002) [25], similar to this current study which found that excessive gingival display significantly affects OHIP-14 domains: functional limitation (*p* < 0.001), physical disability (*p* = 0.003), psychological discomfort (*p* < 0.001), and psychological disability (*p* < 0.001).

Al Sayed et al. reported patient's responses that were quite different from the current study [35]; the most common response for all the OHIP-14 questionnaire 14 questions was *very often* (4), whereas in the current study, the most common response for most of the OHIP-14 questions was *never* (0), apart from Question 5: “feeling self-conscious,” Question 6: “feeling tense” (psychological discomfort), Question 9: “difficult to relax,” and Question 10: “embarrassed” (psychological disability), where the most common response was *occasionally* (2). The striking difference between the responses may be owing to the young age of Al Sayed et al.'s sample, which was 16–18-year-old females, and their sample consisted only of teenagers, where all the emotions are far more heightened than in adults [35], while the present study's sample was 18–40-year-old females. In this current study, a minor positive relationship was found between the extent of gingival display and the psychological aspect of OHIP, meaning that an increase in extent of gingival display leads to a very minute increase in psychological discomfort and psychological disability, indicating that the presence of excessive gingival display affected oral health–related quality of life, but the extent of gingival display did not affect oral health–related quality of life as much.

Within the limitations of this current study, excessive gingival display affects oral health–related quality of life of young Egyptian females, with a minor positive correlation between the extent of gingival display and its effect on the psychological domain. It should be noted that the Egyptian population was less affected by gummy smile than other populations [25, 35], perhaps due to the immense amount of stress the Egyptian citizens experience [37–40] or due to a lower level of patient awareness of oral health–related quality of life [41]. Further research is needed to measure Egyptian's awareness and perception of oral health. In addition, the Egyptian population showed a weak positive correlation, indicating that the presence of excessive gingival display affected the oral health–related quality of life of patients regardless of the amount of gingival display. The findings of this present study help clinicians to understand the extent of the impact of excessive gingival display on the oral health–related quality of life of patients and suggest a need for taking into consideration patients' desires when formulating a treatment plan. The results of this study can be generalized to other populations of similar cultural, demographic, and economic backgrounds.

## 5. Conclusion

Within the limitations of the present study, excessive gingival display significantly affects oral health–related quality of life, particularly the psychological domain of oral health–related quality of life of Egyptian females, with a weak positive correlation with the extent of display. Further studies are required to identify the differences between races regarding societal norms and beauty standards.

## Figures and Tables

**Table 1:** Comparison of the age of the two groups (see S2).

**Age**	**EGD group**	**Non-EGD group**	**p** **value**⁣^∗^
Mean (standard deviation)	27.62 (6.21)	27.06 (6.06)	0.287⁣^∗^
Median	26	25	
Mode	22	23	
Range	18–40	18–39	

⁣^∗^Mann–Whitney *U* test.

**Table 2:** Comparison of OHIP scores between the two groups (see S2).

**OHIP-14 domains (0–8)**	**EGD group**				**Non-EGD group**				
	**Mean (standard deviation)**	**Median**	**Mode**	**Range**	**Mean (standard deviation)**	**Median**	**Mode**	**Range**	**p** **value**⁣^∗^
Functional limitation	0.12 (0.53)	0.0	0	0–4	0.62 (92)	0	0	0–4	<0.001⁣^∗^
Physical pain	0.78 (1.07)	0.0	0	0–4	0.60 (0.78)	0	0	0–3	0.252⁣^∗^
Physical disability	0.97 (1.133)	1.0	0	0–4	0.68 (0.92)	0	0	0–3	0.003⁣^∗^
Psychological discomfort	2.03 (1.22)	2.0	2	0–4	0.55 (0.85)	0	0	0–4	0.001⁣^∗^
Psychological disability	1.42 (1.09)	2.0	2	0–4	0.46 (0.86)	0	0	0–3	0.001⁣^∗^
Social disability	0.68 (1.02)	0.0	0	0–4	0.52 (0.82)	0	0	0–3	0.162⁣^∗^
Handicap	0.37 (0.74)	0.0	0	0–3	0.26 (0.60)	0	0	0–3	0.98⁣^∗^
Total OHIP-14 score	6.37 (3.34)	6	6	0–16	3.68 (2.54)	3	3	0–12	<0.001⁣^∗^

⁣^∗^Mann–Whitney *U* test.

## Data Availability

The data that support the findings of this study are available from the corresponding author upon reasonable request.

## Nomenclature

STROBE: Strengthening the Reporting of Observational Studies in Epidemiology
UNC-15 Probe: University of North Carolina Probe 15 mm
SD: standard deviation
EGD: excessive gingival display
OHRQoL: oral health–related quality of life
OHIP-14: Oral Health Impact Profile-14
OHIP-49: Oral Health Impact Profile-49

## Supporting Information

Supporting Information 1 contains the raw data of the candidates involved in this study.

Supporting Information 2 contains the raw statistical analysis charts and tables before tailoring them into the Results.

## References

[1] Kokich V. O., Kiyak H. A., Shapiro P. A. (1999). Comparing the Perception of Dentists and Lay People to Altered Dental Esthetics. *Journal of Esthetic Dentistry*.

[2] Peck S., Peck L., Kataja M. (1992). The Gingival Smile Line. *The Angle Orthodontist*.

[3] Peck S., Peck L., Kataja M. (1992). Some Vertical Lineaments of Lip Position. *American Journal of Orthodontics*.

[4] Cortellini P., Bissada N. F. (2018). Mucogingival Conditions in the Natural Dentition: Narrative Review, Case Definitions, and Diagnostic Considerations. *Journal of Clinical Periodontology*.

[5] Jacobs P. J., Jacobs B. P. (2013). Lip Repositioning With Reversible Trial for the Management of Excessive Gingival Display: A Case Series. *The International Journal of Periodontics & Restorative Dentistry*.

[6] Hunt O., Johnston C., Hepper P., Burden D., Stevenson M. (2002). The Influence of Maxillary Gingival Exposure on Dental Attractiveness Ratings. *European Journal of Orthodontics*.

[7] Abu Alhaija E. S., Al-Shamsi N. O., Al-Khateeb S. (2011). Perceptions of Jordanian Laypersons and Dental Professionals to Altered Smile Aesthetics. *European Journal of Orthodontics*.

[8] Batra P., Kaur H., Dawar A., Mehta V. (2022). The Threshold of Acceptability of Excessive Gingival Display by Laypersons: A Systematic Review. *International Journal of Esthetic Dentistry*.

[9] Geron S., Atalia W. (2005). Influence of Sex on the Perception of Oral and Smile Esthetics With Different Gingival Display and Incisal plane inclination. *The Angle Orthodontist*.

[10] Malkinson S., Waldrop T. C., Gunsolley J. C., Lanning J. K., Sabatini R. (2013). The Effect of Esthetic Crown Lengthening on Perceptions of a Patient’s Attractiveness, Friendliness, Trustworthiness, Intelligence, and Self Confidence. *Journal of Periodontology*.

[11] Dym H., Pierre R. (2020). Diagnosis and Treatment Approaches to a “Gummy Smile”. *Dental Clinics of North America*.

[12] Elhiny O. A. (2014). Prevalence of Gummy Smile in a Sample of Egyptian Population and Laymen Perception of Its Attractiveness. *Egyptian Orthodontic Journal*.

[13] Locker D. (1988). Measuring Oral Health: A Conceptual Framework. *Community Dental Health*.

[14] Slade G. D., Spencer A. J. (1994). Development and Evaluation of the Oral Health Impact Profile. *Community Dental Health*.

[15] Slade G. D., Spencer A. J. (1994). Social Impact of Oral conditions Among Older Adults. *Australian Dental Journal*.

[16] Oliveira B. H., Nadanovsky P. (2005). Psychometric Properties of the Brazilian Version of the Oral Health Impact Profile-Short Form. *Community Dentistry and Oral Epidemiology*.

[17] Gera A., Cattaneo P. M., Cornelis M. A. (2020). A Danish Version of the Oral Health Impact Profile-14 (OHIP-14): Translation and Cross-Cultural Adaptation. *BMC Oral Health*.

[18] Xin W. N., Ling J. Q. (2006). Validation of a Chinese Version of the Oral Health Impact Profile-14. *Zhonghua Kou Qiang Yi Xue Za Zhi*.

[19] Montero J., Bravo M., Vicente M. P., Galindo M. P., Lopez J. F., Albaladejo A. (2010). Dimensional Structure of the Oral Health-Related Quality of Life in Healthy Spanish Workers. *Health and Quality of Life Outcomes*.

[20] Lahti S., Suominen-Taipale L., Hausen H. (2008). Oral Health Impacts Among Adults in Finland: Competing Effects of Age, Number of Teeth, and Removable Dentures. *European Journal of Oral Sciences*.

[21] Tjan A. H., Miller G. D., The J. G. P. (1984). Some Esthetic Factors in a Smile. *The Journal of Prosthetic Dentistry*.

[22] Rigsbee O. H., Sperry T. P., BeGole E. A. (1988). The Influence of Facial Animation on Smile Characteristics. *The International Journal of Adult Orthodontics and Orthognathic Surgery*.

[23] Elhiny O. (2014). Prevalence of Gummy Smile in a Sample of Egyptian Population and Laymen Perception of it's Attractiveness. *Egyptian Journal of Orthodontics*.

[24] Çetin M. B., Sezgin Y., Akinci S., Bakirarar B. (2021). Evaluating the Impacts of Some Etiologically Relevant Factors on Excessive Gingival Display. *The International Journal of Periodontics & Restorative Dentistry*.

[25] Antoniazzi R. P., Fischer L. S., Balbinot C. E. A., Antoniazzi S. P., Skupien J. A. (2017). Impact of Excessive Gingival Display on Oral Health Related Quality of Life in a Southern Brazilian Young Population. *Journal of Clinical Periodontology*.

[26] Volchansky A., Cleaton-Jones P. E. (1974). Delayed Passive Eruption. A Predisposing Factor to Vincent’s Infection?. *Journal of the DASA*.

[27] Alpiste-Illueca F. (2009). Altered Passive Eruption (APE): A Little-Known Clinical Situation. *Medicina Oral, Patología Oral y Cirugía Bucal*.

[28] Nart J., Carrió N., Valles C. (2014). Prevalence of Altered Passive Eruption in Orthodontically Treated and Untreated Patients. *Journal of Periodontology*.

[29] Andijani R. I., Tatakis D. N. (2019). Hypermobile Upper Lip is Highly Prevalent Among Patients Seeking Treatment for Gummy Smile. *Journal of Periodontology*.

[30] LaFrance M., Hecht M. A., Paluck E. L. (2003). The Contingent Smile: A Meta-Analysis of Sex Differences in Smiling. *Psychological Bulletin*.

[31] Mannaa A. I. (2023). Knowledge and Attitude Toward Esthetic Dentistry and Smile Perception. *Cureus*.

[32] Levi Y. L. A. S., Cota L. V. S., Maia L. P. (2019). Digital Smile Design for Gummy Smile Correction. *Indian Journal of Dental Research*.

[33] Gatto R. C. J., Garbin A., Corrente J., Garbin C. (2017). Self-Esteem Level of Brazilian Teenagers victims of Bullying and Its relation With the Need of Orthodontic Treatment. *RGO-Revista Gaúcha de Odontologia*.

[34] Vig R. G., Brundo G. C. (1978). The Kinetics of Anterior Tooth Display. *The Journal of Prosthetic Dentistry*.

[35] Al Sayed A. A., Alshammari B. Z., Alshammari A. R., Aldajani M. B., Alshammari F. R. (2023). Gummy Smile Prevalence Among Ha’il City Female Young Adults and Its Impact on Quality of Life: A Cross-Sectional Study. *Cureus*.

[36] Hunt R. J., Slade G. D., Strauss R. (1995). Differences between Racial groups in the Impact of oral disorders Among Older adults in North Carolina. *Journal of Public Health Dentistry*.

[37] Fawzy M., Hamed S. A. (2017). Prevalence of Psychological Stress, Depression and Anxiety Among Medical Students in Egypt. *Psychiatry Research*.

[38] Desouky D., Allam H. (2017). Occupational Stress, Anxiety and Depression Among Egyptian Teachers. *Journal of Epidemiology and Global Health*.

[39] Mohamed M. Y., Elbatrawy A. N., Mahmoud D. A. M., Mohamed M. M., Rabie E. S. (2023). Depression and Suicidal Ideations in Relation to Occupational Stress in a Sample of Egyptian Medical Residents. *International Journal of Social Psychiatry*.

[40] Riyad M. A., Ramadan M. E., Alkhadrawy R. A. A. A. (2019). Measurement of Psychological Stress in a Group of Journalists Working in One of the Private Journalism Institutes in Egypt. *Egyptian Journal of Hospital Medicine*.

[41] Moussa A., Ibrahim E., Esmat A., Eissa S., Ramzy M. (2020). An Overview of Oral Health Status, Socioeconomic and Behavioral Risk Factors, and the Pattern of Tooth Loss in a Sample of Egyptian Rural Population. *Bulletin of the National Research Centre*.

