# Supplementary material for: The Psychological Effect of Excessive Gingival Display on Egyptian Females
Source: ScientificWorldJournal. 2025 Oct 23;2025:9996803. doi: 10.1155/tswj/9996803 (PMC12575022; doi:10.1155/tswj/9996803)
Supplement: Supporting Information 2 — contains the raw statistical analysis charts and tables before tailoring them into the Results. [file 9996803.f2.docx]

| **Ranks** | | | | |
| --- | --- | --- | --- | --- |
|  | EGDorControl | N | Mean Rank | Sum of Ranks |
| Functional limitation | 1 | 320 | 371.47 | 118869.50 |
|  | 2 | 320 | 269.53 | 86250.50 |
|  | Total | 640 |  |  |
| Physical Pain | 1 | 320 | 313.03 | 100169.00 |
|  | 2 | 320 | 327.97 | 104951.00 |
|  | Total | 640 |  |  |
| Physical disability | 1 | 320 | 300.32 | 96103.00 |
|  | 2 | 320 | 340.68 | 109017.00 |
|  | Total | 640 |  |  |
| Psychological discomfort | 1 | 320 | 216.63 | 69322.00 |
|  | 2 | 320 | 424.37 | 135798.00 |
|  | Total | 640 |  |  |
| Psychological disability | 1 | 320 | 243.29 | 77851.50 |
|  | 2 | 320 | 397.71 | 127268.50 |
|  | Total | 640 |  |  |
| Social Disability | 1 | 320 | 311.77 | 99766.00 |
|  | 2 | 320 | 329.23 | 105354.00 |
|  | Total | 640 |  |  |
| Handicap | 1 | 320 | 311.95 | 99824.50 |
|  | 2 | 320 | 329.05 | 105295.50 |
|  | Total | 640 |  |  |
| total | 1 | 320 | 244.47 | 78231.50 |
|  | 2 | 320 | 396.53 | 126888.50 |
|  | Total | 640 |  |  |
| Age | 1 | 320 | 312.74 | 100078.00 |
|  | 2 | 320 | 328.26 | 105042.00 |
|  | Total | 640 |  |  |

| **Test Statistics^a^** | | | | | | | | | |
| --- | --- | --- | --- | --- | --- | --- | --- | --- | --- |
|  | Functional limitation | Physical Pain | Physical disability | Psychological discomfort | Psychological disability | Social Disability | Handicap | total | Age |
| Mann-Whitney U | 34890.500 | 48809.000 | 44743.000 | 17962.000 | 26491.500 | 48406.000 | 48464.500 | 26871.500 | 48718.000 |
| Wilcoxon W | 86250.500 | 100169.000 | 96103.000 | 69322.000 | 77851.500 | 99766.000 | 99824.500 | 78231.500 | 100078.000 |
| Z | -9.583 | -1.145 | -3.008 | -14.802 | -11.451 | -1.399 | -1.653 | -10.447 | -1.064 |
| Asymp. Sig. (2-tailed) | .000 | .252 | .003 | .000 | .000 | .162 | .098 | .000 | .287 |
| a. Grouping Variable: EGDorControl | | | | | | | | | |

| **Test Statistics^a^** | | | | | | | | | |
| --- | --- | --- | --- | --- | --- | --- | --- | --- | --- |
|  | Functional limitation | Physical Pain | Physical disability | Psychological discomfort | Psychological disability | Social Disability | Handicap | total | Age |
| Mann-Whitney U | 34890.500 | 48809.000 | 44743.000 | 17962.000 | 26491.500 | 48406.000 | 48464.500 | 26871.500 | 48718.000 |
| Wilcoxon W | 86250.500 | 100169.000 | 96103.000 | 69322.000 | 77851.500 | 99766.000 | 99824.500 | 78231.500 | 100078.000 |
| Z | -9.583 | -1.145 | -3.008 | -14.802 | -11.451 | -1.399 | -1.653 | -10.447 | -1.064 |
| Asymp. Sig. (2-tailed) | .000 | .252 | .003 | .000 | .000 | .162 | .098 | .000 | .287 |
| a. Grouping Variable: EGDorControl | | | | | | | | | |


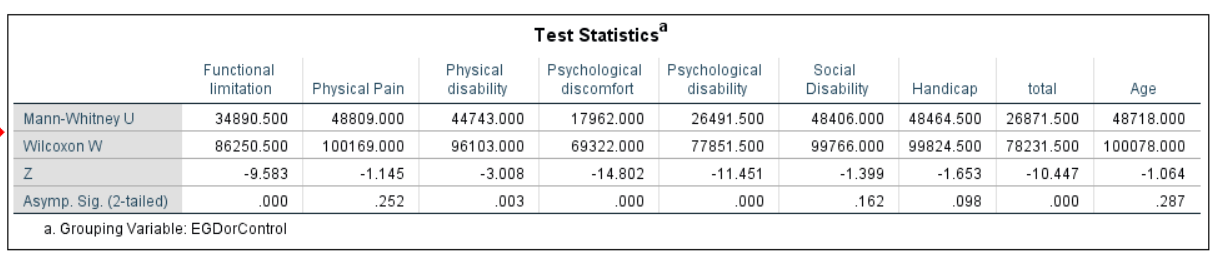


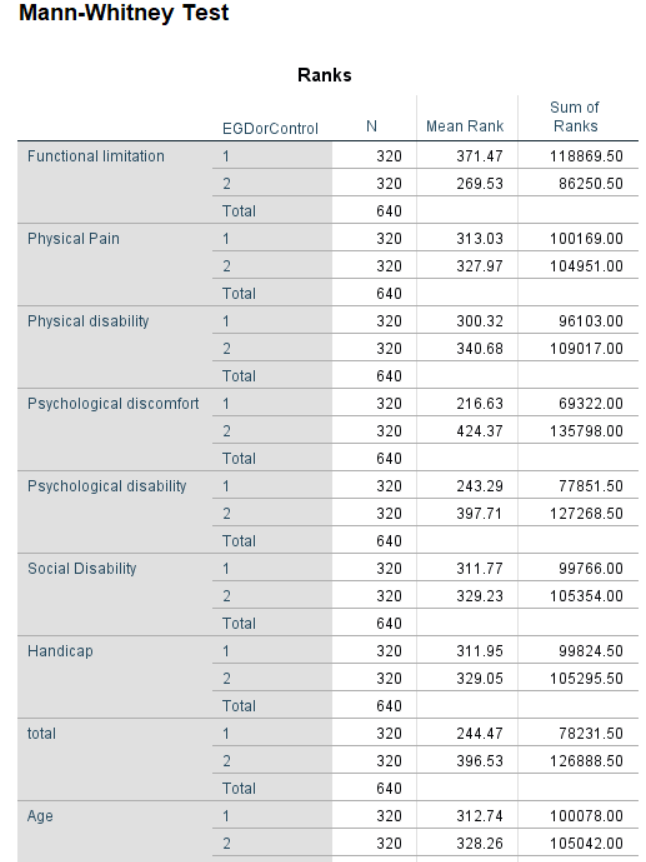


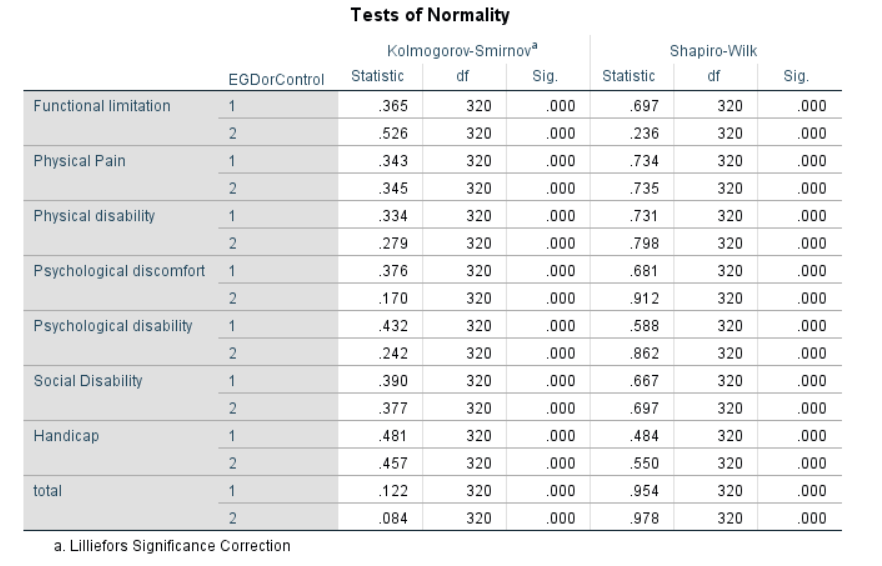


Control group:

| **Descriptive Statistics** | | | | | | | | |
| --- | --- | --- | --- | --- | --- | --- | --- | --- |
|  | N | Range | Minimum | Maximum | Mean | | Std. Deviation | Variance |
|  | Statistic | Statistic | Statistic | Statistic | Statistic | Std. Error | Statistic | Statistic |
| Functional limitation | 320 | 4 | 0 | 4 | .62 | .051 | .916 | .839 |
| Physical Pain | 320 | 3 | 0 | 3 | .60 | .044 | .782 | .611 |
| Physical disability | 320 | 3 | 0 | 3 | .68 | .052 | .922 | .850 |
| Psychological discomfort | 320 | 4 | 0 | 4 | .55 | .047 | .848 | .718 |
| Psychological disability | 320 | 3 | 0 | 3 | .46 | .048 | .859 | .738 |
| Social Disability | 320 | 3 | 0 | 3 | .52 | .046 | .823 | .677 |
| Handicap | 320 | 3 | 0 | 3 | .26 | .034 | .600 | .360 |
| total | 320 | 12 | 0 | 12 | 3.68 | .142 | 2.537 | 6.436 |
| Age | 320 | 21 | 18 | 39 | 27.06 | .339 | 6.063 | 36.761 |
| Valid N (listwise) | 320 |  |  |  |  |  |  |  |

| **Statistics** | | | | | | | | | | |
| --- | --- | --- | --- | --- | --- | --- | --- | --- | --- | --- |
|  | | Functional limitation | Physical Pain | Physical disability | Psychological discomfort | Psychological disability | Social Disability | Handicap | total | Age |
| N | Valid | 320 | 320 | 320 | 320 | 320 | 320 | 320 | 320 | 320 |
|  | Missing | 0 | 0 | 0 | 0 | 0 | 0 | 0 | 0 | 0 |
| Mean | | .62 | .60 | .68 | .55 | .46 | .52 | .26 | 3.68 | 27.06 |
| Median | | .00 | .00 | .00 | .00 | .00 | .00 | .00 | 3.00 | 25.00 |
| Mode | | 0 | 0 | 0 | 0 | 0 | 0 | 0 | 3 | 23 |
| Std. Deviation | | .916 | .782 | .922 | .848 | .859 | .823 | .600 | 2.537 | 6.063 |
| Variance | | .839 | .611 | .850 | .718 | .738 | .677 | .360 | 6.436 | 36.761 |
| Range | | 4 | 3 | 3 | 4 | 3 | 3 | 3 | 12 | 21 |
| Minimum | | 0 | 0 | 0 | 0 | 0 | 0 | 0 | 0 | 18 |
| Maximum | | 4 | 3 | 3 | 4 | 3 | 3 | 3 | 12 | 39 |
| Percentiles | 25 | .00 | .00 | .00 | .00 | .00 | .00 | .00 | 2.00 | 23.00 |
|  | 50 | .00 | .00 | .00 | .00 | .00 | .00 | .00 | 3.00 | 25.00 |
|  | 75 | 1.00 | 1.00 | 1.00 | 1.00 | 1.00 | 1.00 | .00 | 5.00 | 31.00 |

Gummy Group :

| **Statistics** | | | | | | | | | | |
| --- | --- | --- | --- | --- | --- | --- | --- | --- | --- | --- |
|  | | FunctionalLimitation | PhysicalPain | PhysicalDisability | PsychologicalDiscomfort | PsychologicalDisability | SocialDisability | Handicap | total | Age |
| N | Valid | 320 | 320 | 320 | 320 | 320 | 320 | 320 | 320 | 320 |
|  | Missing | 0 | 0 | 0 | 0 | 0 | 0 | 0 | 0 | 0 |
| Mean | | .12 | .78 | .97 | 2.03 | 1.42 | .68 | .37 | 6.37 | 27.62 |
| Median | | .00 | .00 | 1.00 | 2.00 | 2.00 | .00 | .00 | 6.00 | 26.00 |
| Mode | | 0 | 0 | 0 | 2 | 2 | 0 | 0 | 6 | 22 |
| Std. Deviation | | .530 | 1.074 | 1.133 | 1.224 | 1.094 | 1.019 | .743 | 3.339 | 6.214 |
| Variance | | .281 | 1.154 | 1.284 | 1.498 | 1.198 | 1.038 | .552 | 11.149 | 38.619 |
| Range | | 4 | 4 | 4 | 4 | 4 | 4 | 3 | 16 | 22 |
| Minimum | | 0 | 0 | 0 | 0 | 0 | 0 | 0 | 0 | 18 |
| Maximum | | 4 | 4 | 4 | 4 | 4 | 4 | 3 | 16 | 40 |
| Sum | | 38 | 251 | 310 | 648 | 455 | 219 | 117 | 2038 | 8838 |
| Percentiles | 25 | .00 | .00 | .00 | 1.00 | .00 | .00 | .00 | 4.00 | 23.00 |
|  | 50 | .00 | .00 | 1.00 | 2.00 | 2.00 | .00 | .00 | 6.00 | 26.00 |
|  | 75 | .00 | 2.00 | 2.00 | 3.00 | 2.00 | 1.00 | .00 | 9.00 | 32.00 |
